# Neutrophil–lymphocyte ratio as a predictor of adverse outcome in patients with community‐acquired pneumonia: A systematic review

**DOI:** 10.1002/hsr2.630

**Published:** 2022-05-02

**Authors:** Sandip Kuikel, Nibesh Pathak, Sagar Poudel, Sital Thapa, Shiva Lal Bhattarai, Gajendra Chaudhary, Kundan Raj Pandey

**Affiliations:** ^1^ Maharajgunj Medical Campus Tribhuvan University Institute of Medicine Kathmandu Nepal; ^2^ Department of Internal Medicine Tribhuvan University Institute of Medicine Kathmandu Nepal

**Keywords:** adverse outcome, CAP, NLR, pneumonia, predictor

## Abstract

**Background:**

Community‐acquired pneumonia (CAP) is the acute infection of lung tissue in an immunocompetent who acquired it from the community. Its incidence and mortality are significant and require a marker to predict the severity and mortality in these patients. Neutrophil–lymphocyte ratio (NLR) is a simple, cheap, and easy‐to‐use marker and this study describes its role in predicting the adverse outcome in patients with CAP.

**Methods:**

PubMed, EMBASE, and Google Scholar were used to search for related studies on February 8, 2021. A total of 186 articles were retrieved upon detailed searching in the databases and search engines. After a series of removing duplicate articles, title and abstract screening, and full‐text review; nine articles were found eligible and included in the study. The data from each article were collected in MS Excel and the findings were summarized in this manuscript.

**Results:**

The total number of patients analyzed in this systematic review is 3340. The mean age of the patient in the included studies ranged from 61 to 90.4 years. All studies had adverse outcomes as the endpoint of the study, which included in‐hospital mortality or intensive care unit (ICU) admission or deterioration from medium and low risk to high risk or 30 days' mortality. The prevalence of endpoint ranged from 5.8% to 44.8%. NLR with a cutoff value of more than 10 was shown to predict mortality compared to C‐reactive protein levels, white blood cell count, neutrophil count, lymphocyte level, Pneumonia Severity Index (PSI) level, PSI class, procalcitonin, and CURB‐65 (Confusion, Respiratory rate, Blood pressure, 65 years of age and older) in most of the studies.

**Conclusion:**

NLR is a simple, easily measured yet promising marker for predicting outcomes in patients with CAP.

## 1 INTRODUCTION

Pneumonia is a form of acute respiratory infection involving the lungs.^1^ Pneumonia is one of the major causes of hospitalization in the United States. It accounted for more than 800,000 hospitalizations and more than 400,000 emergency department visits in 2014 in the United States.^2^ In 2010, out of reported 52.8 million deaths globally, lower respiratory tract infection accounted for about 3.4 to 2.8 million deaths.^3^ Pneumonia is caused by a various number of infectious agents that are bacteria or fungi or viruses. The commonly attributed organisms in adults are *Streptococcus pneumonia*, *Haemophilus influenzae*, respiratory syncytial virus, and *Pneumocystis jiroveci* in patients with HIV/AIDS. Other less common organisms causing pneumonia include *Mycoplasma pneumonia*, *Chlamydophila pneumonia*, *Legionella* sp., and *Pseudomonas aeruginosa*.^2^

Pneumonia has been classified into four types, namely, community‐acquired pneumonia (CAP), hospital‐acquired pneumonia, healthcare‐associated, and ventilator‐associated pneumonia.^4^ CAP is an acute infection of lung tissue in an immunocompetent patient who was not recently hospitalized, or had been hospitalized only for less than 48 h and acquired it from the community.^4^, ^5^ The incidence and mortality of CAP are higher at extremes of age accounting for 5.15–7.06 cases per 1000 persons per year in adults.^4^ CAP is one of the leading causes of death globally.^6^ It causes about 102,000 deaths per year in the United States alone with mortality of 13% at 1 month, 23.4% at 6 months, and 30.6% at 12 months.^7^

Pneumonia Severity Index (PSI) and Confusion, Respiratory rate, Blood pressure, 65 years of age and older (CURB‐65) are commonly used tools among many scoring systems for assessing the severity and predicting mortality in patients with CAP.^8^ However, none of the scoring systems is ideal and some scores are cumbersome to be used in day‐to‐day clinical practice. Inflammatory biomarkers in the blood‐like C‐reactive protein (CRP) and procalcitonin may improve the prognostic accuracy of these scores.^9^, ^10^ However, these two biomarkers are not always reliable,^11^ thus arising the need to identify a biomarker that is reliable, cheap, and easy to use. One of the studied markers includes neutrophil–lymphocyte ratio (NLR), which is an easily measurable index. It is the ratio of absolute neutrophil count to absolute lymphocyte count. Under pathological stress, the number of neutrophils increases, whereas the number of lymphocytes decreases. This systematic review tries to define the role of NLR in determining the outcome in patients with CAP.

## 2 METHODS

### 2.1 Searching strategy

This study was done in accordance with the Preferred Reporting Items for Systematic Reviews and Meta‐Analyses (PRISMA) statement. A search strategy was developed and used for literature review in two of the major databases: PubMed and EMBASE. The literature review was done by searching for the remaining articles in the reference to related articles. The keywords used for the search in major databases were “Community acquired pneumonia,” “CAP,” “neutrophils,” “lymphocytes,” and “ratio,” and “survival,” “mortality,” or “prognosis.” Only human filter was applied in our search. The search was conducted on February 8, 2021, and articles published up to the search date were included in the study. Our systematic review was not prospectively registered with any of the international Systematic Review Registers.

### 2.2 Selection of studies

Six items (PICOTS) were used to define the question for our systematic review of NLR as a prognostic factor, based on CHARMS (checklist for critical appraisal and data extraction for systematic reviews of prediction modeling studies):

- Population: Patient admitted with a diagnosis of CAP, diagnosis made either radiologically or clinically.
- Index prognostic factor: NLR was the single biomarker reviewed for its prognostic value.
- Other conventional prognostic factors of interest were age, sex, smoking status, obesity, diabetes, CURB‐65, and PSI.
- Outcome: In‐hospital mortality, 30 days' mortality, adverse outcome (ICU admission).
- Timing: NLR had to be measured at the time of admission.
- Setting: NLR measurement was studied in in‐hospital care settings to provide prognostic information about patients diagnosed with CAP; this information may be useful for healthcare professionals treating and managing such patients.

Predefined inclusion and exclusion criteria were used to identify suitable studies. Only studies that explored the role of NLR as a prognostic factor for CAP were included. To be included, studies had to investigate outcomes such as mortality or ICU admission or degradation from low risk and medium risk (MR) to high risk (HR).

The following exclusion criteria were applied:

- review articles, research protocols,
- case series/case reports,
- symposium/conference proceedings, commentaries/editorials/letters, views/opinions,
- full‐text unavailable,
- not in English.

Title and abstract screening were done using Covidence by two reviewers. A third author resolved conflicts between two reviewers. All the studies that qualified the predefined inclusion criteria were screened for full‐text review and this process too was done by two reviewers. Overall agreement between the two reviewers was very good (70%–80%). A third author resolved the conflicts between the two authors.

### 2.3 Data extraction

Data extraction was done in MS Excel version 2016. Data extraction was done by two reviewers, followed by rechecking of the extracted data by a third reviewer. Data extraction template was made on MS Excel and the following data were extracted from each study: author of the study, year of publication, study design, study setting, patient number (with gender distribution), the mean age of study population, variables, measured outcomes of patients, NLR cutoff, prevalence of the outcomes, prognostic estimates (HR or OR) or sensitivity or specificity or area under the ROC curve (AUC) (whichever was available), and main conclusions.

## 3 RESULTS

### 3.1 Study selection

**Figure 1:**
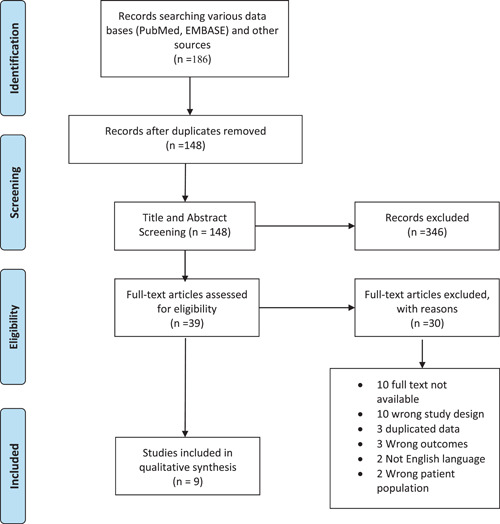
PRISMA flow diagram showing the study retrieval process.

In our search, 55 studies were obtained from PubMed, 93 studies from EMBASE and 38 studies from a literature review from other sources like references of related articles. After the removal of 38 duplicate articles, 148 articles were eligible for subsequent screening. Upon title and abstract screening, 39 articles were eligible for full‐text screening, amongst which 30 articles were excluded for various reasons. A total of nine articles were included in the qualitative synthesis of this systematic review. The PRISMA flow diagram (Figure 1) depicts the study retrieval process used.

### 3.2 Study and patient characteristics

A total of nine studies from six different countries (one with a study in which country name is not mentioned) were included in the qualitative synthesis. Two studies included patients in the emergency department, five included admitted patients in the general ward, two studies included patients in ICU, and one study included patients from both general ward and ICU. The total number of patients analyzed in this systematic review was 3340, and out of them, 1878 (56.22%) were male. The sample size of the included studies ranged from 100 to 1549. Almost all of the studies had a sample size of less than 500, except one,^12^ which included 1549 patients. The mean age of the patient in the studies included ranged from 61 to 90.4 years. All studies had adverse outcomes as an endpoint of the study, the adverse outcome being ICU admission or deterioration from MR and low risk to HR or in‐hospital mortality or 30 days' mortality. The prevalence of endpoint ranged from 5.8% to 44.8% (Table 1).

**Table 1:** Study and patient characteristics of included studies

Author	Year	Country	Study design	Clinical setting	Endpoint	Sample size	Male (percentage)	Age	Cutoff of NLR	Prevalence (number) of endpoint
de Jager et al.^13^	2012	Netherlands	Cohort study	ED	Adverse event (mortality/adverse events)	395	61 (15.44%)	63 ± 16	10	12.7% (50)
					In‐hospital mortality				10	5.8% (23)
Avci and Pericnek ^14^	2020	Turkey	Prospective cross‐sectional	ED	ICU admission	206	128 (62.13%)	68.34 ± 16.52	N/A	8.7% (18)
					30‐day mortality				N/A	22.8% (47)
Cataudella et al.^15^	2017	Italy	Prospective	In patient	30‐day mortality	195	120 (61.54%)	80.3	11.2	24% (47)
									13.4	
Curbelo et al.^16^	2017	Spain	Prospective cohort	In patient	30‐day mortality	154	89 (57.79%)	12(90.4 ± 501)	10	7.79% (12)
								+142(74.5 ± 16.1)		
Masbang et al.^17^	2019	Philippines	Prospective cross‐sectional	In patient	Predicting HR from lr and MR	280	123 (43.92%)	68.9 ± 18.54	10.24	13.9% (39)
Ozmen et al.^18^	2016	Turkey	Retrospective cohort	ICU	Short‐term mortality (<30 days)	143	83(58%)	70 ± 12	N/A	
					Mortality 180 days after ICU admission					
					ICU mortality					18.88% (27)
Postma et al.^12^	2016	N/A	Randomized crossover trial	In patient	30‐day mortality	1549	996 (64.3%)	70 (58–79)	Median = 10.4	5.9% (92)
					90‐day mortality					
Yang et al.^19^	2017	China	Retrospective cross‐sectional	In patient	In‐hospital mortality	318	211 (66.35%)	61	Median in patient who died (11.96; IQR: 7.26–30.68)	7.2% (23)
									Median in patient who did not die (4.19; IQR: 2.39–7.52)	
									7.12	
Kaya ^20^	2018	Turkey	Prospective	In patient	Mortality	67	42 (62.69%)	66.8 ± 12.5		0% (0)
				ICU	Mortality	33	25 (75.76%)	75.3 ± 10.3	Dead NLR = 13.5 ± 9	44.8% (15)
									Survived NLR = 7.9 ± 6.8	

Abbreviations: ED, emergency department; HR, high risk; ICU, intensive care unit; IQR, interquartile range; LR, low risk; MR, moderate risk; N/A, not available; NLR, neutrophil–lymphocyte ratio.

### 3.3 NLR as a predictor of adverse outcome

In this review, we evaluated if NLR could predict adverse outcome of patients with CAP. In the nine included studies, the association between NLR and adverse outcome was evaluated and the association was significant in all of the included studies.^12^, ^13^, ^14^, ^15^, ^16^, ^17^, ^18^, ^19^, ^20^ However, this association was confirmed by multivariate analysis in only one study.^12^ NLR was shown to have high mortality prediction compared to CRP levels, WBC count, neutrophil count, lymphocyte level, PSI level, PSI class, procalcitonin, and CURB‐65 in most of the studies.^13^, ^14^, ^15^, ^16^, ^17^, ^19^ However, a study done by Kaya et al.^20^ concluded that NLR is not superior to the commonly used scoring system (PSI, CURB‐65) in estimating mortality. NLR had a sensitivity of 56.4%–78.26% and specificity of 51.61%–66.8% in predicting adverse outcome when its cutoff was taken to be 10.^13^, ^14^, ^16^, ^17^ However, the sensitivity and specificity of NLR was 100% and 77.7%, respectively, when a cutoff of 11.2 was taken.^15^ The sensitivity and specificity were 91.49% and 83.78%, respectively when the cutoff was 13.4.^15^ The rate of mortality increased on increasing NLR^19^, ^20^ (Table 2).

**Table 2:** Prognostic estimates effect of NLR and conclusion of each study

Author	CURB‐65 (2–5)	End point	Cutoff of NLR	Sensitivity	Specificity	Prognostic effect estimates (HR, OR)	AUC	Variables	Conclusion
de Jager et al.^13^	123	Adverse event (mortality/adverse events)	10	74 (95% CI: 59.66–85.37)	53.33 (95% CI: 47.91–58.69)	N/A	N/A	Age, gender, comorbidities, medications, pathogens, CRP level	NLR better‐predicted mortality compared to CRP levels, WBC count, neutrophil count, and lymphocyte count
		In‐hospital mortality	10	78.26 (95% CI: 56.3–92.54)	51.61 (95% CI: 46.4–56.8)	N/A	0.701		
Avci and Perincek^14^	N/A	30‐day mortality	N/A	N/A	N/A	N/A	0.577 (95%CI 0.501‐0.650)	Age, smoking, comorbidities, complication, clinical parameters	NLR showed low 30‐day mortality estimation accuracy than PSI class, PSI scores and procalcitonin
Cataudella et al.^15^	175	30‐day mortality	11.2	100 (95% CI: 92.45–100)	77.7 (95% CI: 70.14–84.13)	N/A	0.94	Age, sex, CURB‐65, PSI, comorbidities	NLR predicted mortality better than PSI, CURB‐65, CRP, and WBC count
			13.4	91.49 (95% CI: 76.84–89.33)	83.78 (95% CI: 76.84–89.33)	N/A			
Curbelo et al.^16^	128	30‐day mortality	10	63.6 (95% CI: 35.4–84.3)	65 (95% CI: 56.8–72.4)	OR = 1.04 (1.0–1.1)	0.88 (95% CI: 0.79–0.98)	Age, sex, vaccination, comorbidities, PSI, CURB‐65, CRP, procalcitonin, proadrenomedullin	NLR was not inferior to proadrenomedullin and significantly better than other biomarkers
Masbang et al.^17^	N/A	Predicting HR from LR and MR	10.24	56.4	66.8	N/A	0.726	Age, gender, comorbidity, smoking, symptoms, vital signs, radiologic finding	NLR predicts CAP severity more than WBC count. NLR can better predict HR from LR and MR
Ozmen et al.^18^	N/A	30‐day mortality	N/A	N/A	N/A	HR = 1.04 (0.99–1.01)	0.64 (0.49–0.79)	Age, gender, comorbidity, smoking, biochemistry, ventilation, ABG finding	Higher NT‐pro BNP values (above 2000 pg/ml) and NLR can be used to predict pneumonia severity and higher NLR on admission to ICU has a higher risk of 180‐day mortality
		Mortality 180 days after ICU admission	N/A	N/A	N/A	HR = 1.04 (1.01–1.07)	0.63 (0.52–0.74)		
		ICU mortality	N/A	N/A	N/A	N/A	0.60(0.46–0.75)		
Postma et al.^12^	N/A	30‐day mortality	Median = 10.4	N/A	N/A	OR = 1.19 (95% CI: 1.02–1.38)	N/A	Age, sex, comorbidities, clinical parameters, mortality scores	NLR had a moderate bivariate association with mortality, but was not statistically significant when added to the model with either PSI or CURB‐65
		90‐day mortality	N/A	N/A	N/A	OR = 1.18 (95% CI: 1.05–1.32)	N/A		
Yang et al.^19^	67	In‐hospital mortality (in patient)	Median in patient who died (11.96; IQR: 7.26–30.68)	82.61	72.2	N/A	0.799	Age, sex, PCT, CRP, comorbidities, PSI, CURB‐65	NLR is a simple promising marker for predicting in‐hospital mortality
		In‐hospital mortality (ICU)	Median in patient who did not die (4.19; IQR : 2.39–7.52)	N/A	N/A	N/A	N/A		
			Cutoff = 7.12						
Kaya^20^	N/A	In‐patient mortality	N/A	N/A	N/A	N/A	N/A	Age, sex, comorbidities, PSI, CURB, laboratory values	NLR can be used in estimating mortality, but is not superior to the commonly used scoring system (PSI, CURB‐65)
		ICU Mortality	Dead NLR = 13.5 ± 9	N/A	N/A	N/A	0.743 (95% CI: 0.627–0.860)		
			Survived NLR = 7.9 ± 6.8	N/A	N/A	N/A			

Abbreviations: AUC, area under the ROC curve; CI, confidence interval; CRP, C‐reactive protein; CURB‐65, Confusion, Respiratory rate, Blood pressure, *65* years of age and older; HR, high risk; HR, hazard ratio; ICU, intensive care unit; IQR, interquartile range; LR, low risk; MR, medium risk; N/A, not available; NLR, neutrophil–lymphocyte ratio; NT‐proBNP, N‐terminal (NT)‐prohormone B‐type natriuretic peptide; OR, odds ratio; PCT, procalcitonin; PSI, Pneumonia Severity Index; WBC, white blood cell.

The study done by Postma et al.^12^ analyzed 1549 CAP patients admitted to non‐ICU wards and investigated the value of the NLR alone or in conjunction with existing scoring systems to predict 30‐day mortality in CAP, and explored associations with 90‐day all‐cause mortality, length of stay, microbial etiology, and occurrence of complicated pneumonia. The studied population had a median length of stay of 6 days and a 30‐day mortality of 5.9%. Bivariate analysis showed that NLR was associated with mortality, odds ratio (OR): 1.19 (95% confidence interval [CI]: 1.02–1.38) per 10 units increase, but when it was added to PSI or CURB‐65 score, NLR did not significantly improve prediction models (*p* = 0.18 and *p* = 0.11 respectively) and there was no significant difference in AUC for PSI (0.752 vs. 0.761, *p* = 0.10) and CURB‐65 (0.698 vs. 0.709, *p* = 0.246). NLR was associated with complicated pneumonia (adjusted OR: 1.24 (95% CI: 1.03–1.49)) per 10 units increase, and with the occurrence of pneumococcal (adjusted OR: 1.31 (95% CI: 1.17–1.47)) or bacterial etiology (adjusted OR: 1.27 (95% CI: 1.15–1.41)), but there was no statistically significant association with 90‐day mortality or length of stay.^12^

The study by de Jager et al.^13^ also investigated the value of the NLR by taking adverse events or mortality as the endpoint. NLR levels (mean ± SD) were significantly higher in nonsurvivors (23.3 ± 16.8) than in survivors (13.0 ± 11.4). The receiver‐operating characteristic (ROC) curve for NLR predicting mortality showed an area under the curve (AUC) of 0.701, and the AUC for the neutrophil count, WBC count, lymphocyte count, and CRP level were 0.681, 0.672, 0.630, and 0.565, respectively. Taking NLR cut‐off point of 10 showed a sensitivity of 74% and a specificity of 53.33%. It was concluded that NLR better‐predicted mortality compared to CRP levels, WBC count, neutrophil count, and lymphocyte count.

The findings of the study by Avci et al.^14^ were not in congruence with other articles included in this review. In their study, they studied 206 patients diagnosed with CAP and evaluated comorbidities, arterial blood gas, serum electrolytes, liver–renal functions, complete blood count, NLR, CRP, PSI, CURB‐65, and procalcitonin. NLR (AUC 0.58) had the lowest 30‐day mortality estimation in contrast to procalcitonin (AUC: 0.65), PSI class (AUC: 0.81), and PSI score (AUC: 0.86), which indicated that PSI class, PSI score, and procalcitonin had statistically significant higher 30‐day mortality prediction.

The accuracy and predictive value for 30‐day mortality of traditional scores and NLR were compared by Cataudella et al.^15^ in their study. In this study, no deaths occurred in participants with an NLR of less than 11.12; the 30‐day mortality was 30% in those with an NLR between 11.12% and 13.4%, while those with an NLR between 13.4 and 28.3 had 30 days mortality of 50%. All participants, in this study, with an NLR greater than 28.3 died within 30 days. It showed that NLR predicted 30‐day mortality and prognosis was better predicted than PSI, CURB‐65, CRP, and white blood cell count. The results of the study recommend early discharge of individuals with an NLR of less than 11.12, short‐term in‐hospital care for those with an NLR between 11.12 and 13.4, middle‐term hospitalization for those with an NLR between 13.4 and 28.3, and admission to a respiratory intensive care unit for those with an NLR greater than 28.3.

Curbelo et al.^16^ investigated the association between concentrations of several inflammatory markers and mortality of CAP patients. The association of outcome variables (mortality at 30 and 90 days) with CRP, procalcitonin, proadrenomedullin, copeptin, white blood cell, lymphocyte count percentage (LCP), neutrophil count percentage (NCP), and NLR were studied. The study showed that patients who died during follow‐up had higher levels of procalcitonin, copeptin, proadrenomedullin, lower levels of LCP, and higher levels of NCP and NLR. Thus, NLR and NCP at admittance and during early‐stage evolution had good diagnostic power.

The study by Masbang et al.^17^ aimed to establish the predictive value of WBC count and NLCR in classifying CAP and assess the predictive value of WBC count and NLR. The sensitivity and specificity of WBC and NLR were determined for the following: (1) between CAP low risk (LR) versus CAP MR and CAP HR and (2) between CAP LR and CAP MR versus CAP HR. The mean average of NLR per risk was 5.4, 8.6, and 16.1 for LR, MR, and HR, respectively. Higher NLR was associated with higher risk; thus, NLR could be used to stratify patients to low risk, MR, and HR.

The findings of the study by Ozmen et al.,^18^ Yang et al.^19^ and Kaya et al.^20^ had similar findings. A study by Ozmen et al.^18^ showed that NLR could be used to predict pneumonia severity and a higher NLR on admission to ICU had a higher risk of 180 days of mortality. In the study by Yang et al.,^19^ the median NLR in patients who died was higher compared to the median NLR in patients who did not die (11.96; IQR: 7.26–30.68 vs. 4.19; IQR: 2.39–7.52). In the study by Kaya et al.,^20^ the mean NLR in CAP patients in ICU was lower in patients who survived compared to patients who died (7.9 ± 6.8 vs. 13.5 ± 9); thus, NLR can be used in estimating mortality. However, NLR was not found to be superior to other commonly used scoring systems like PSI and CURB‐65.

## 4 DISCUSSION

In this review, we evaluated the role of NLR in predicting adverse outcomes in patients with CAP. There was strong evidence for its use in predicting the adverse outcome in the included studies.^12^, ^13^, ^14^, ^15^, ^16^, ^17^, ^18^, ^19^, ^20^ Most of the studies^12^, ^13^, ^14^, ^15^, ^16^, ^17^, ^18^, ^19^ showed the superiority of NLR to existing scoring systems like CURB‐65 and PSI and conventionally used biomarkers like procalcitonin, and proadrenomedullin, CRP levels, and WBC counts. However, NLR when used in conjunction with PSI or CURB‐65 did not yield statistical significance to predict the adverse outcome than when PSI or CURB‐65 was used alone.^12^ The highest sensitivity of NLR in predicting adverse outcomes were observed when the cutoff value of 11.2 was used, but the highest specificity was noted with the cutoff value of 13.4.^15^ However, a clear definition of cutoff value remains to be undetermined.

Total and differential leukocyte count is the most common blood test done in cases of an infectious disease.^21^ Neutrophils and lymphocytes are the main mediators of inflammation. The ratio of two provides an insight into the disease severity and helps predict the outcome of patients with CAP.^22^ The value of NLR, both as a prognostic marker and a marker of response to treatment, in other inflammatory conditions and different tumors is already proven.^23^, ^24^, ^25^, ^26^, ^27^, ^28^ Its prognostic value is shown by Lugg et al.^23^ and Scilla et al.^24^ in non‐small cell carcinoma, by Seo et al.^25^ in idiopathic sensory neural hearing loss, by Pirozzolo et al.^26^ in esophageal carcinoma, by Liu et al.^27^ in patients receiving chemotherapy for lung cancer, and by Ni et al.^28^ in patients with sepsis. The clinical role of this easy‐to‐obtain inflammatory biomarker is also shown in ischemic stroke,^29^, ^30^ cerebral hemorrhage,^31^, ^32^ and major cardiac events.^33^ Similarly, it is a significant prognostic marker in predicting adverse outcomes in patients with CAP as per the studies included in this review.^12^, ^13^, ^14^, ^15^, ^16^, ^17^, ^18^, ^19^, ^20^

However, a lot of confounding factors may play role in determining mortality in patients with CAP like an etiological agent, antimicrobial susceptibility, age of the patient, and comorbidities.^34^ Although NLR has good sensitivity and specificity in predicting adverse outcomes, these confounding factors should be taken into account. Thus, further studies are required to determine its independent and combined role with PSI and CURB‐65 in predicting adverse outcomes in patients with CAP. If supported by a large number of evidence, NLR would have more advantage over more conventionally used predictors like mid‐regional adrenomedullin,^18^, ^35^ procalcitonin,^36^ and CRP levels.^37^ The use of NLR alone in clinical practice to predict the adverse outcome in patients with CAP is not justifiable given the data available in the existing literature and as summarized in our review. It could be combined with other scoring systems or other conventional biomarkers to improve its prognostic power, but further studies are required to further support its use.

This review summarizes the findings of different studies in predicting the adverse outcome of patients with CAP using NLR. This may help in‐patient management by stratifying patients with high NLR to intensive care unit and with low NLR to general in‐hospital management. This is supported by the fact that patients with high NLR are shown to have high mortality; thus, they may require early critical care support. There are some limitations to this study, such as quantitative synthesis of data could not be done (because of the variability of reporting prognostic effect estimates). Other limitation of the study includes the fact that NLR can be easily affected by different conditions like comorbidities, which was taken into account by only a few studies and included studies that had wide variation in reporting the statistical prognostic effect estimates for adverse outcomes.

## 5 CONCLUSION

NLR is a simple, easily measured, yet promising marker for predicting outcomes in patients with CAP. Its value, either alone or in conjunction with other biomarkers and scoring systems, must be further investigated.

## AUTHOR CONTRIBUTIONS

**Sandip Kuikel**: Conceptualization, literature review, protocol development, title and abstract review, full‐text review, data extraction, manuscript writing, revision, and submission. **Nibesh Pathak** and **Sagar Poudel**: Protocol development, title and abstract review, and manuscript writing. **Sital Thapa**: Full‐text review and manuscript writing. **Shiva Lal Bhattarai**: Data extraction and manuscript writing. **Gajendra Chaudhary** and **Kundan Raj Pandey**: Manuscript writing and proof‐reading and revisions.

## CONFLICT OF INTEREST

The authors declare no conflicts of interest.

## TRANSPARENCY STATEMENT

The lead author (manuscript guarantor) affirms that this manuscript is an honest, accurate, and transparent account of the study being reported; that no important aspects of the study have been omitted; and that any discrepancies from the study as planned (and, if relevant, registered) have been explained.

## Data Availability

The data are accessible via referenced articles. Any further data regarding the article can be made available upon reasonable request to the corresponding author.
